# Endometrial scratch vs no intervention in egg donation cycles: the ENDOSCRATCH trial protocol

**DOI:** 10.1186/s12884-020-02958-0

**Published:** 2020-05-30

**Authors:** Alexandra Izquierdo, Laura de la Fuente, Katharina Spies, Jennifer Rayward, Lourdes López, David Lora, Alberto Galindo

**Affiliations:** 1 ProcreaTec Fertility Clinic, Madrid, Spain; 2 Human Reproduction Unit, Department of Obstetrics and Gynecology, University Hospital 12 de Octubre, Madrid, Spain; 3 Clinical Research Unit (imas12-CIBERESP). University Hospital 12 de Octubre, Madrid, Spain; 4 Fetal Medicine Unit – Maternal and Child Health and Development Network (Red SAMID-RD12/0026/0016). Department of Obstetrics and Gynecology. University Hospital 12 de Octubre. 12 de Octubre Research Institute (imas12), Complutense University of Madrid, Madrid, Spain

**Keywords:** Endometrial scratching, Endometrial injury, In vitro fertilization, Recurrent implantation failure, Egg donation, Hysteroscopy, Endometrial receptivity

## Abstract

**Background:**

The effects of endometrial scratching (ES) on embryo implantation have been studied for many years. Several studies have shown better outcomes when performed on patients undergoing intrauterine insemination and in vitro fertilization (IVF) cycles, but many other reports have not been able to find these differences. As far as cycles with donor eggs are concerned, reported evidence is scarce. Our aim in this trial is to determine if ES is useful for those patients undergoing IVF cycles with donor eggs, in order to assure a greater homogeneity in embryo quality and endometrial preparation.

**Methods:**

This single centre randomized controlled trial will include patients undergoing an egg donation cycle, meeting the inclusion criteria and who accept to participate in the study. Once informed consent is signed, patients will be randomly allocated to the study arm (group A) and then receive ES in the luteal phase of the cycle prior to embryo transfer, or the control arm (group B) without any intervention. All cycle data will be collected and analyzed to obtain the clinical pregnancy and the live birth rates in the two groups.

**Discussion:**

Several studies have tried to determine the effectiveness of an ES in IVF cycles, but it is still unclear due to the heterogeneity of these reports. The aim of this study is to determine if there are differences in clinical pregnancy rate and live birth rate in egg donor cycles, when comparing an ES performed in the preceding luteal phase versus no intervention, given that embryo quality and endometrial preparation protocols will be comparable.

**Trial registration:**

Ethical approval of version 2.0 of this trial was obtained on the 13th January 2017. It was retrospectively registered on the 5th April 2017 as the ENDOSCRATCH Trial (NCT03108157) in ClinicalTrials.gov.

## Background

Embryo implantation remains one of the main challenges in assisted reproduction. Relevant improvements have been accomplished in reproductive medicine: different protocols for controlled ovarian stimulation and endometrial preparation, embryo culture with time-lapse technologies, embryo pre-implantational genetic testing and endometrial genetic assessment for implantation potential. Despite the fact that these changes have led to increasing pregnancy rates in the last few years, the implantation process is still inefficient, as it remains around 30% of all embryos replaced [1], and it is not yet totally understood.

The endometrium is a dynamic tissue with a complex architecture that undergoes several changes during the menstrual cycle which have an important impact on embryo implantation. Endometrial scratching (ES) is a simple procedure aiming to create a mild endometrial injury that has been proposed to improve the embryo-endometrium dialogue. Different authors have attributed this improvement to the effects of different cytokines and growth factors involved in an acute endometrial inflammatory process [2], the enhancement of new vascularization and decidualization [3], the improvement of endometrial maturation [4], and the promotion of endometrial gene expression that may lead to a better synchrony between the embryo and the endometrium [5].

A Cochrane Review by Lensen et al. which includes 9 randomized controlled trials (RCTs), tried to determine the effects of ES in intrauterine insemination (IUI) cycles or in spontaneous conception cycles [6], suggesting a potential benefit of this technique. However, evidence was low or very low graded due to important limitations of the studies considered (study design and low number of patients included). Senocak et al. [7] found a better clinical pregnancy rate (CPR) and ongoing pregnancy rate (OPR) when scratching was performed the cycle prior to the insemination (OR 2,29, 95%IC:1.14–5.05), but a previous meta-analysis by Vitagliano et al. [8] found similar differences when it was performed during the stimulation cycle (OR 2,04, *p* < 0,001).

Regarding in vitro fertilization (IVF) cycles, several studies have tried to determine whether an endometrial injury performed in the cycle preceding the embryo transfer could enhance embryo implantation. Barash et al. [9] reported for the first time a two-fold increase in pregnancy rate in patients that had undergone multiple ES before the IVF cycle, compared to those patients who had no ES performed.

Since then, many authors have tried to determine ES effects after controlled ovarian stimulation (COS), but while some of them have found an increase in pregnancy rates [9–12], many others have been unable to find such differences [13–18]. The main limitation in reaching a conclusion is that most of these are underpowered observational studies, with a low number of patients included, with differences in timing (luteal or follicular phase from the preceding or same cycle), number of ES (one, two or more procedures), type of catheter and different stimulation protocols. It is important to note that those studies that have found some positive effects of ES have included patients with implantation failures [12, 19] whereas those that included patients in their first or second IVF cycle were unable to find any differences [15, 16]. It is also relevant that some studies included as control patients, those who had undergone a hysteroscopy prior to the IVF cycle and, even if an ES was not performed in these patients, we may assume that the endometrium was exposed to some “damage” as well [4]. Another study included a cervical biopsy for those patients included in the control group, what cannot be really considered as “placebo” [17].

A systematic review conducted by Potdar et al. [20] including 7 studies with 2062 patients, found a three-fold increase in pregnancy rates in those patients that received ES. Similar results were also found some time later by a Cochrane Review by Nastri et al. [21] with moderate-quality evidence, signaling the need for well-designed trials without uterine instrumentation in the control group, stratification for implantation failure and the necessity to report live birth rates. This review also showed that endometrial injury on the day of oocyte retrieval decreased live birth (RR 0.31, 95% CI 0.14 to 0.69) and clinical pregnancy (RR 0.36, 95% CI 0.18 to 0.71).

All these studies were conducted after COS, but there is only one retrospective study in patients receiving embryos from donor eggs, and who have not undergone ovarian stimulation [22]. When comparing egg donation cycles to other IVF treatments, we find two main differences: the first one is that embryo quality is presumably optimal, since all embryos come from donor eggs, avoiding the confusion factor of embryo quality according to maternal issues (age, BMI, polycystic ovaries, low ovarian reserve …) and the second difference is that all patients receive hormone replacement therapy with a homogeneous preparation of the endometrium, avoiding different hormonal environments caused by diverse responses to controlled ovarian stimulation in IVF.

Our main purpose is to determine whether a mild endometrial injury (ES) performed the cycle prior to the embryo transfer can help endometrial receptivity and thus, synchronization between the embryo and the endometrium, enhancing the implantation process in egg donation cycles. ES is usually performed with a plastic biopsy catheter, 3 mm in diameter, (Pipelle de Cornier, Laboratoire CCD, France) “scratching” the four walls of the uterine cavity and is performed in an out-patient setting, without anesthesia, under transabdominal ultrasound guidance.

This trial tries to minimize the confounding factors by selecting only egg recipients and normal sperm partners, avoiding the possible detrimental effect of embryo quality on pregnancy rates. In addition, all recipients will receive a substituted cycle to prepare the endometrium for the embryo implantation, reducing the variability among protocols.

### Hypothesis

If ES performed during the cycle preceding embryo transfer is an effective procedure in itself, it should show its benefit in egg recipients, the ideal population where embryos are of maximum quality and where the endometrium is homogenously prepared.

## Methods

### Study objective

The main objective of the ENDOSCRATCH trial is to determine if there are differences in pregnancy rates in egg donor IVF treatments when comparing patients receiving an ES before endometrial preparation for embryo transfer and those who will not receive any intervention.

There are other studies whose endpoint was pregnancy rate after spontaneous conception, IUI and IVF or after implantation failures, but our intention is to minimize confounding factors in terms of embryo quality and endometrial hormonal preparation comparing egg donor IVF cycles.

### Study design

This is a single-centre prospective RCT, fully conducted at Procreatec Fertility Clinic in Madrid, starting January 2017 to December 2019 to evaluate the effectiveness of an endometrial biopsy (scratching) before endometrial preparation, during the luteal phase of the previous cycle versus the conventional treatment protocol for egg donation IVF without endometrial biopsy.

Those patients undergoing an egg donation cycle that meet the inclusion criteria will be invited to participate in this study. We will obtain informed consent (IC) from all patients, according to the guidelines of the Ethics Committee. Once patients have accepted the study and signed the IC, they will be allocated to each treatment arm, according to the randomization protocol. Those patients included in Group A will undergo an ES during the luteal phase of the cycle prior to the embryo transfer. Those patients assigned to Group B will follow the conventional protocol without ES (Additional File 1). A total of 352 patients will be recruited.

All information regarding patients, assignment, treatment protocol and results will be included in our database to conduct the statistical analysis.

The primary endpoint of this RCT is CPR, which will be determined via vaginal ultrasound at approximately 6 weeks pregnancy. Secondary endpoints are biochemical pregnancy rate (BPR), ongoing pregnancy rate (OPR), implantation rate (IR), miscarriage rate (MR), live birth rate (LBR) and cumulative pregnancy rate (CumPR). Biochemical pregnancy will be determined by blood β-hCG levels over 10 mUI/ml 12 days after the embryo transfer. Ongoing pregnancy will be assessed via ultrasound beyond 12 weeks of pregnancy. IR will be determined by the ratio between the number of gestational sacs and the number of replaced embryos. Early miscarriage will be assessed if pregnancy stops before the 12th week of pregnancy. Late miscarriage will be assessed if pregnancy stops between the 12th and the 24th week of pregnancy. Live birth will be determined by direct contact with patients, who will report the pregnancy outcome. CumPR will be evaluated 12 months after randomization for all patients.

### Study population: inclusion and exclusion criteria

All patients undergoing egg donation treatments at ProcreaTec Fertility Clinic are eligible for the study.

Patients will be included if they meet the following inclusion criteria:

- Age between 18 and 50 years.
- Primary or secondary infertility.
- Patients undergoing an IVF protocol with donor eggs
- Normal uterine cavity (transvaginal ultrasound scan)
- Patients with endometrial polyps can be included as long as polypectomy is performed at least 2 months before the treatment cycle
  Patients will be excluded if:
- There is a severe male factor (less than 2 million sperms per ml)
- They have uterine anomalies such as uterine fibroids that impact the cavity, Mullerian malformations or severe adenomyosis
- They have unilateral or bilateral hydrosalpinx
- They have undergone a previous ES or hysteroscopy (at least 1 month before the randomization)
- Pre-implantation genetic testing cycles

### Sample size calculation

The average CPR after embryo transfer in egg donor IVF cycles is 60% at our centre. Based on previous studies, where the difference in CPR for IVF cycles varied between 10 to 30% [9, 11, 12, 19, 20, 23–25], we estimated that a 15% difference in CPR would be clinically relevant. According to that percentage, a total of 332 patients will be needed to detect a 15% difference between the two groups, with 80% statistical power and two-sided alpha of 0,05. Considering a 5% dropout rate, we will include 176 patients per study arm, 352 patients in total.

### Recruitment, consent and randomization

Patients starting egg donor IVF cycles that fulfill inclusion criteria will be offered participation. If they agree, IC will be signed and they will be assigned to a treatment group by the patient’s doctor. A randomization chart will be obtained by a web-based randomization program using random blocks (randomization.com). Since patients in the study group will receive an intervention and those in the control group will not (no placebo intervention will be performed), blinding is not possible for patients nor for physicians.

### Interventions

Once patients have accepted the study and signed the IC, they will be allocated to each treatment group with random allocation 1:1, according to the randomization chart. Patients allocated to Group A will be given a specific date for the ES to be performed between 5 to 10 days before their period starts and the endometrial preparation begins. The ES will be done in an out-patient setting. A speculum will be inserted in the vagina and, after cervix disinfection with iodine solution, an endometrial biopsy catheter (Pipelle de Cornier, Laboratoire CCD, France) will be introduced into the uterine cavity to scratch the four walls. The procedure will be carried out under transabdominal ultrasound guidance to help insertion and the correct position of the catheter before and during the procedure. Patients allocated to Group B will directly start endometrial preparation with their menses.

In order to prepare the endometrium for embryo reception, all patients will receive hormonal replacement therapy with oral (Estradiol Valerate 2 mg every 8 h) or transdermal estrogen (Estradiol Valerate 200 mcg every 48 h). After 10 to 12 days of preparation, an endometrial scan will be performed to check the endometrial thickness. Those patients with a thin lining (less than 6 mm) will receive additional estrogen dose (Estradiol Valerate 2 mg every 12 h vaginally). Patients with an endometrial thickness over 6 mm will continue with the same protocol until the day of the egg retrieval of the donor.

The day when eggs will be fertilized, we will perform a last endometrial scan and a blood test to check estradiol and progesterone levels. That night, all patients will start the progesterone treatment (Micronized progesterone pessaries 400 mg every 12 h). Embryo transfer will take place 3 to 5 days later and patients will continue with the hormone treatment until the day of the pregnancy test, 12 days after the embryo transfer. Transfer procedure will be cancelled if endometrial thickness is under 6 mm, or if hormone levels reveal signs of premature ovulation.

Those patients with ovarian activity would also receive medication for ovarian quiescence to avoid any follicle growth during the endometrial preparation (Decapeptyl® or Orgalutran®).

### Follow-up

We will follow the development of the cycle, from the moment the donor is assigned until we have performed a pregnancy test, and if positive, then the ultrasound confirmation for pregnancy 10 to 12 days later. We will follow all pregnancies to determine LBR as well as possible pregnancy and delivery complications. All patients will be followed for a 12-month period to determine the CumPR.

### Data collection and analysis

All study variables will be collected from patients included in the trial, from ProcreaTec clinical records, according to the information required in the data collection form. Each doctor will include relevant information in the patients’ clinical record and the principal investigator will be responsible for collecting and managing the information. Any adverse events will be reported by responsible doctors and managed by the principal investigator.

Baseline characteristics of patients included will be analyzed as follows. Qualitative variables will be described using mean and standard deviation, non-normal variables will be summarized using median and 25 and 75% centiles. Qualitative variables will be described using frequency distribution.

The main outcome, CPR, and secondary outcomes, BPR, OPR, MR, IR, LBR and CumPR for each group will be analyzed with Chi-Squared test or Fisher’s exact test. Efficacy of the treatment will be described as absolute and relative frequencies, together with the association strength by raw risk ratio (RR) with 95% confidence intervals. In addition, a general linear model, with a log link and binomial distribution, will be used to estimate the strength of association between primary and secondary outcomes adjusted by independent variables. A subgroup analysis will be performed to assess possible differences between patients based on whether or not they have had previous implantation failure.

Results will be presented as RR and 95% confidence intervals. Statistical significance will be 0,05 (5% both sides α error) for all comparisons. Statistical analysis will be done using Stata 13 for Windows (StataCorp LP, Texas).

### Results communication

Once final results are obtained, they will be submitted to those journals focused on assisted reproduction techniques.

## Discussion

Embryo implantation is the limiting factor to attain pregnancy. It occurs in about 30% of all conceptions and it remains a major challenge for all assisted reproduction treatments. Both patients and specialists have great concern when the treatment fails and many times the only step which is not fully understood is the embryo-endometrium crosstalk.

The improvement of implantation potential has been studied in different ways: optimization of COS protocols, freeze-all programs, preimplantational genetic testing of the embryos, immunological approaches and determination of the endometrial implantation window. These treatments and procedures have all tried to maximize the chances of embryo implantation. The apposition, adhesion and invasion of the endometrium are defining processes that the embryo has to be able to achieve, while the endometrium has to become receptive and interact with an appropriate immunologic reaction.

ES has been proposed by several authors as a simple, easy and cost-effective technique to improve the endometrial receptivity, enhancing embryo implantation.

As a recent study shows, despite the lack of robust evidence, many specialists tend to recommend ES for recurrent implantation and many others even in the first or second attempt as well [26]. Nevertheless, some authors have reported an important discomfort [19] and additional costs for the patient, which should also be taken into account, as well as the potential detrimental effects on the endometrium [27].

This RCT will try to clarify whether ES can improve pregnancy rates in egg donor IVF treatments, being thus beneficial for those patients, or if there is only a specific group of patients for whom it may be specially indicated. This will be analysed in women 18 to 50 years (the most common age range for women to undergo an egg donation cycle), in order to assess whether the impact of ES depends on the patient’s age. The main strength for this study will be the number of recruited patients, as well as the homogeneity in endometrial preparation protocols and embryo quality.

## Supplementary information

**Additional file 1.** Participant timeline figure.

## Data Availability

The datasets used and/or analyzed during the current study will be available from the corresponding author on reasonable request.

## Abbreviations

IUI: Intrauterine insemination
IVF: In Vitro Fertilization
COS: Controlled ovarian stimulation
ES: Endometrial scratching
IC: Informed consent
RCT: Randomized controlled trial
CPR: Clinical pregnancy rate
BPR: Biochemical pregnancy rate
OPR: Ongoing pregnancy rate
IR: Implantation rate
MR: Miscarriage rate
LBR: Live birth rate
CumPR: Cumulative pregnancy rate
RR: Risk ratio
OR: Odds ratio
