# Supplementary figures and images for: Endometrial scratch vs no intervention in egg donation cycles: the ENDOSCRATCH trial protocol
Source: BMC Pregnancy Childbirth. 2020 May 30;20:333. doi: 10.1186/s12884-020-02958-0 (PMC7260784; doi:10.1186/s12884-020-02958-0)

Additional File 1. The ENDOSCRATCH Trial Protocol (NCT03108157) Participant timeline

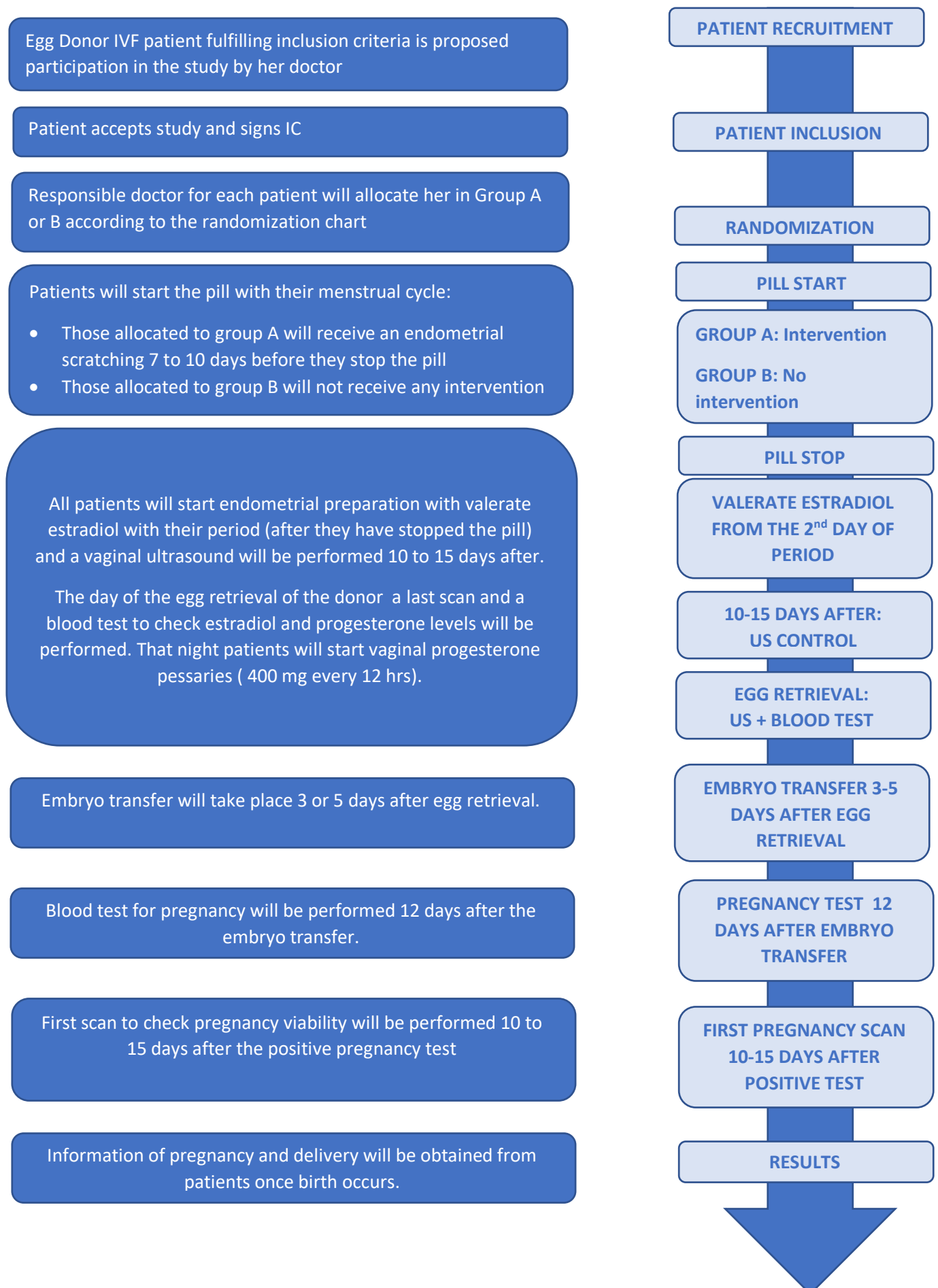

Supplement: Supplementary file 1 — Additional file 1. Participant timeline figure. [file 12884_2020_2958_MOESM1_ESM.pdf]
